# Different Fertilizers Applied Alter Fungal Community Structure in Rhizospheric Soil of Cassava (*Manihot esculenta* Crantz) and Increase Crop Yield

**DOI:** 10.3389/fmicb.2021.663781

**Published:** 2021-11-10

**Authors:** Jie Cai, Jie Zhang, Yun Ding, Shan Yu, Hongxin Lin, Zhanqi Yuan, Kaimian Li, Wenjun Ou, Songbi Chen

**Affiliations:** ^1^Key Laboratory of Ministry of Agriculture for Germplasm Resources Conservation and Utilization of Cassava, Tropical Crops Genetic Resources Institute, Chinese Academy of Tropical Agricultural Sciences, Haikou, China; ^2^College of Horticulture, Hainan University, Haikou, China; ^3^Soil and Fertilizer and Resources and Environment Institute, Jiangxi Academy of Agricultural Sciences, Nanchang, China

**Keywords:** cassava (*Manihot esculenta*), fertilization, high-throughput sequencing, rhizospheric soil, fungal community structure

## Abstract

Soil microbes play an important role in the ecosystem and have a relationship with plant growth, development, and production. There are only a few reports on the effects of planting patterns of cassava on the microbial community structure in the rhizospheric soil. Here, we investigated the effects of different fertilization on the microbial community structure in the cassava rhizospheric soil. SC205 cultivar was used in this study as the experimental material. Compound fertilizer (CF) and reduced fertilizer (RF) were applied to the soil prior to planting. Soil samples were collected before harvest, and fungi were analyzed using IonS5^TM^XL sequencing platform. Results showed that CF and RF treatments significantly increased cassava yield. Amplicon sequencing result indicated that the fungi richness in rhizospheric soil of cassava was increased after CF was applied, and the diversity was decreased. However, the fungal diversity and richness were decreased in rhizospheric soil after RF was applied. The most dominant fungal phylum was Ascomycota, which increased after fertilization. In addition, the abundance of beneficial fungi such as *Chaetomium* increased after fertilization, while that of pathogenic fungi such as *Fusarium solani* was decreased. The composition of the fungal community in rhizospheric soil with CF and RF applied was similar, but the richness and diversity of fungi were different. Canonical correspondence analysis (CCA) indicates there was a positive correlation between soil nutrition and fungal community structure. Overall, our results indicate that fertilization alters the fungal community structure of cassava rhizospheric soil, such that the abundance of potentially beneficial fungi increased, while that of potentially pathogenic fungi decreased, thereby significantly promoting plant growth and yield of cassava. Thus, during actual production, attention should be paid to maintain the stability of cassava rhizospheric soil micro-ecology.

## Introduction

Cassava (*Manihot esculenta* Crantz) is the staple food in the tropics and sub-tropics, which has high starch content, drought tolerance, high soil acidity tolerance, and strong adaptability and is water efficient (Li et al., 2001). In China, cassava is mainly used as an industrial raw material and feed. The demand for cassava is growing in recent decades. However, the area for cassava cultivation is limited. Scientists have paid more attention to improving quality and increasing yield by developing excellent varieties through crossbreeding and molecular breeding to satisfy the rising demand for cassava. In cassava cultivation, fertilizers are normally used to promote plant growth and increase yield (Zheng et al., 2015; Liang et al., 2019). But long-term excessive and unscientific methods of fertilization resulted in a series of ecological problems, including soil contamination, changed soil microbial community structure, and also damaged crop plants growth, development, and production yield (Pavlidis et al., 2020). Therefore, the application of reduced chemical fertilizers or organic fertilizers should be carried out for cassava cultivation in the future.

Soil microbes play key roles in agricultural ecosystem (Singh et al., 2006; Chaparro et al., 2012; Siciliano et al., 2014; Sterkenburg et al., 2015; Sun et al., 2015; Nie et al., 2018), especially fungi. Fungi can improve and promote plant nutrition acquisition by decomposing soil organic matter (SOM) (Chen et al., 2019). As a consequence, the quantity, richness, and diversity of rhizospheric fungi affect plant growth, development, and crop yield (Gao et al., 2019; Wang et al., 2020). Recently, studies have shown that fertilization alters the rhizospheric fungal community structure (Luo et al., 2015; Yu et al., 2015, 2017; Wang et al., 2017). Under sustainable fertilizers management, beneficial fungi increased, which inhibited pathogenic bacteria and promoted plant growth (Tan et al., 2017; Wang et al., 2020). In contrast, some fungi are plant pathogens (Boas et al., 2016; Braganca et al., 2016). For example, anthracnose, caused by *Colletotrichum*, is a devastating soil-borne disease of cassava that seriously affects the growth and yield of cassava (Obilo et al., 2010). These findings indicate that changes in fungal community structure correlate with fertilization.

At present, several studies have focused on the effect of different fertilizers on promoting growth, improving quality, and increasing the yield of cassava (Zhang et al., 2008; Liang et al., 2019). There are few reports focus on the richness and diversity of cassava rhizospheric soil microbes after fertilization (Xu et al., 2016; Lin et al., 2020). However, there is no report hitherto regarding the effects of reduced application of chemical fertilizers on fungal community structure in cassava rhizospheric soil. In this study, we implemented high-throughput sequencing technology to analyze changes in fungal community structure in rhizospheric soil of cassava under different fertilization and investigated the effects on yield of cassava and its fungal community structure. We studied the effect of fertilization on fungal community structure and their correlation with soil nutrition and crop yield to provide a theoretical basis for promoting growth, improving quality, and increasing yield of cassava and developing high-efficiency cultivation under scientific fertilizer management.

## Materials and Methods

### Description of Study Site and Materials

The experiment was performed in the field at the Nanchang experimental station of Chinese Cassava Agro-technology Research System at Fuzhou, Jiangxi Province, China, in 2019 (28°22′51.63″N, 116°49′11.33″E), a typical acidic red soil region and cassava production region in China. The cassava cultivar South China 205 (SC205) was selected as the material in this study, which is widely used in China as a parent in cassava breeding.

### Soil Physicochemical Properties Analysis

The soil pH was measured by using glass electrode pH meter, in 1:5 soil/water (w/v) suspension (Lu, 2000). After oxidation using K_2_Cr_2_O_7_, the amount of organic matter in soil was measured by titration method (Lu, 2000). Alkali-hydrolyzable nitrogen concentration in soil was measured by diffusion method (Lu, 2000). Available phosphorus in soil was determined by hydrochloric acid-ammonium fluoride method (Lu, 2000). Available potassium concentration in soil was measured by ammonium acetate extraction method.

### Experimental Design and Sample Collection

Compound fertilizer (N:P_2_O_5_:K_2_O: 15:15:15, 1,500 kg ha^–1^, CF) and reduced fertilizer (N:P_2_O_5_:K_2_O: 11.25:7.5:15, 1,500 kg ha^–1^, RF) were the treatments in this study. The experiment was designed as a random block with three experimental replicates. The planted district had nine blocks; each block measured 4 m × 5 m, which had five rows, 10 plants per row. The distance of each replicate was 100 cm, and planting management was the same as for the field.

According to the sample collecting method, soil samples were harvested in the “S” shape. The whole root of cassava was completely excavated with a sampling shovel, and the root was tapped; the sample soil closely adhering to the root was collected and then passed through a 1-mm sieve and stored at –80°C for microbial analysis.

### Yield Parameters of Cassava

The number of tuberous root, stem diameter, plant height, and yield of each treatment were recorded and analyzed.

### DNA Extraction, Amplicon Generation, PCR Products Mixing, and Purification and Sequencing

Microbial DNA from the soil samples was extracted using the cetyltrimethyl ammonium bromide (CTAB)/sodium dodecyl sulfate (SDS) method (Cai et al., 2011). The fungal 18S rRNA and ITS genes of distinct regions were amplified by PCR (98°C for 1 min, followed by 30 cycles of denaturation at 98°C for 10 s, annealing at 50°C for 30 s, elongation at 72°C for 30 s, and final extension at 72°C for 5 min) using a specific primer (Supplementary Table 1) with the barcode. PCR products were mixed in equidensity ratios. The mixture of PCR products was purified with the GeneJET^TM^ Gel Extraction Kit (Thermo Fisher Scientific). Sequencing libraries were generated using Ion Plus Fragment Library Kit 48 reactions (Thermo Fisher Scientific) following manufacturer’s recommendations. The library quality was assessed on the Qubit@ 2.0 Fluorometer (Thermo Fisher Scientific). The library was sequenced on an Ion S5^TM^ XL platform, and 400 bp/600 bp single-end reads were generated.

### Sequencing Data Analysis

Single-end reads were assigned to samples based on their unique barcode and truncated by cutting off the barcode and primer sequence. Quality filtering on the raw reads was performed under specific filtering conditions to obtain the high-quality clean reads according to the Cutadapt quality controlled process (Martin, 2011). Chimera sequences were detected and removed using UCHIME Algorithm (Edgar et al., 2011). Operational taxonomic units (OTUs) were clustered with 97% similarity by Uparse software (Uparse version 7.0.1001).^1^ In order to study the phylogenic relationship of different OTUs, multiple sequence alignments were conducted using the MUSCLE software (Version 3.8.31).^2^

Community richness and diversity indexes were calculated with QIIME (Version 1.7.0) and displayed with R software (Version 2.15.3), including Observed-species, Chao1, Shannon, Simpson, ACE, and Good-coverage. Principal coordinate analysis (PCoA) was displayed by WGCNA package, stat packages, and ggplot2 package in R software (Version 2.15.3). Venn diagram was prepared using Venn diagram to analyze overlapped and unique OTUs. Heatmap figures were generated using the Vegan packages in R to analyze the composition of the fungal community. For CCA, the CCA function in the vegan package is used for the sorting analysis, and the r2 and p describing every environmental factor’s influence on the distribution of the genus were obtained by envfit function. FunGuild is a fungal environmental function database.

## Results

### Soil Nutritional Status of Cassava Rhizospheric Soil Under Different Treatments

The characteristics of the soil after fertilization of cassava are shown in Table 1. The soil pH value was acidic (pH value from 4.10 to 4.38). Compared to without fertilizer applied (CK) treatment, only the available K of CF and RF was significantly increased (*p* ≤ 0.05). Furthermore, the available N and SOM of CF were significantly decreased (*p* ≤ 0.05), but the available P of CF and RF displayed no changes.

**TABLE 1:** The physical and chemical nature of the rhizospheric soil.

Parameters	Treatments		
	CK	CF	RF
Soil pH	4.25	4.10	4.38
Available N (mg kg^–1^)	110.54 ± 1.71 a	96.96 ± 1.21 b	109.98 ± 5.29 a
Available P (mg kg^–1^)	128.63 ± 16.40 a	122.12 ± 24.58 a	126.93 ± 11.64 a
Available K (mg kg^–1^)	25.87 ± 1.51 b	32.33 ± 2.09 a	31.37 ± 2.0 a
SOM (g kg^–1^)	17.28 ± 0.70 a	15.98 ± 0.24 b	17.26 ± 0.59 a

*CK, without fertilizer applied; CF, compound fertilizer applied; RF, reduced fertilizer applied; SOM, soil organic material.*

*Different letters in a row indicate significant differences determined by Kruskal–Wallis test (p ≤ 0.05, n = 3); data are means ± standard errors.*

### Yield Parameters of Cassava

Yield and yield parameters were analyzed to investigate the relationship between different fertilizations and crop yields. Compared to CK treatment, CF and RF treatments significantly increased the number of tuberous roots by 63.5 and 85.7%, stem diameter by 37.2 and 39.2%, plant height by 56.7 and 60.7%, and yield by 50.4 and 56.8%, respectively (Table 2, *p* ≤ 0.05).

**TABLE 2:** The yield and its parameters of cassava under different fertilizations.

Treatments	Number of tuberous roots	Stem diameter (mm)	Plant height (cm)	Yield (kg ha^–^^1^)
CK	6.3 ± 0.18 b	17.75 ± 0.96 b	105.50 ± 6.41 b	9,990.60 ± 87.10 b
CF	10.3 ± 0.68 a	24.36 ± 1.06 a	165.30 ± 9.48 a	15,033.67 ± 123.41 a
RF	11.7 ± 1.57 a	24.70 ± 1.27 a	169.50 ± 10.37 a	15,678.33 ± 173.07 a

*CK, without fertilizer applied; CF, compound fertilizer applied; RF, reduced fertilizer applied.*

*Different letters in a row indicate significant differences determined by Kruskal–Wallis test (p ≤ 0.05, n = 3), data are means ± standard errors.*

### α-Diversity of Fungi in the Rhizospheric Soil of Cassava

**FIGURE 1:**
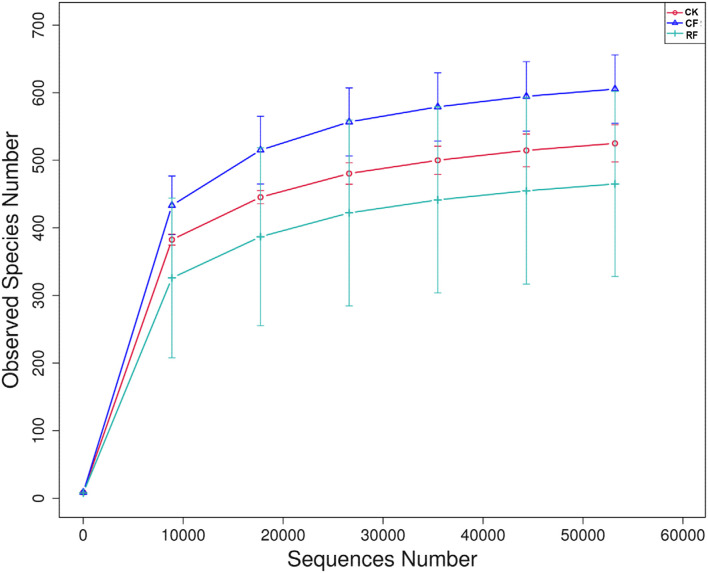
Rarefaction curves for all of the soil samples. CK, CF, and RF indicate without fertilizer applied, with compound fertilizer applied, and reduced fertilizer applied, respectively.

The collected soil samples were assigned to high-throughput sequencing using the Ion S5^TM^ XL platform, and the sequencing results are shown in Table 3. The coverage of all samples was above 99.90%, and the rarefaction curve of each sample had already approached a saturation plateau (Figure 1), indicating that the sequencing library had reached saturation and the results truly reflected the sample condition.

**TABLE 3:** High-throughput sequencing results and α-diversity index of cassava rhizospheric soil samples.

Sample name	Reads	OTUs	Shannon	Simpson	Chao1	ACE	Goods_ coverage
CK	80,066	525 (491, 558)	6.366 (5.956, 6.597)	0.967 (0.949, 0.977)	555.437 (501.684, 620.308)	552.985 (503.143, 608.028)	0.999
CF	80,100	605 (544, 668)	6.288 (6.171, 6.442)	0.964 (0.95, 0.972)	629.764 (573.812, 688.276)	629.51 (571.047, 687.592)	0.999
RF	80,068	465 (310, 643)	5.631 (3.809, 6.719)	0.925 (0.83, 0.974)	486 (339, 670.5)	491.66 (352.211, 666.453)	0.999

*CK, without fertilizer applied; CF, compound fertilizer applied; RF, reduced fertilizer applied; Reads, the optimized sequences; OTU, operational taxonomic unit.*

*The numbers within parentheses are the lower and upper limits in statistics of the corresponding data, respectively.*

The clean reads per sample ranged from 80,066 to 80,100. The OTU per sample ranged from 465 to 605. With CF applied, the number of OTUs of rhizospheric soil fungi showed an overall increasing trend. The Ace and Chao1 also displayed an increasing trend, indicating that the richness of CF applied soil fungi increased with fertilizer application. However, the Shannon and Simpson indexes showed descending trends, indicating the diversity of fungi of cassava rhizospheric soil decreased with fertilizer application. Compared to applied CK and CF, the number of OTUs of rhizospheric soil fungi after RF was applied showed an overall decreasing trend, including the Ace, Chao1, Shannon, and Simpson indexes which showed descending trend indicating that the richness and diversity of soil fungi were lowest among them.

### Fungal Community Composition in the Rhizospheric Soil of Cassava

Analysis of fungal community structure showed that the fungus richness of cassava rhizospheric soil significantly increased after fertilization. Additionally, the fungal proportion of rhizospheric soil was significantly different in differently treated soils.

**FIGURE 2:**
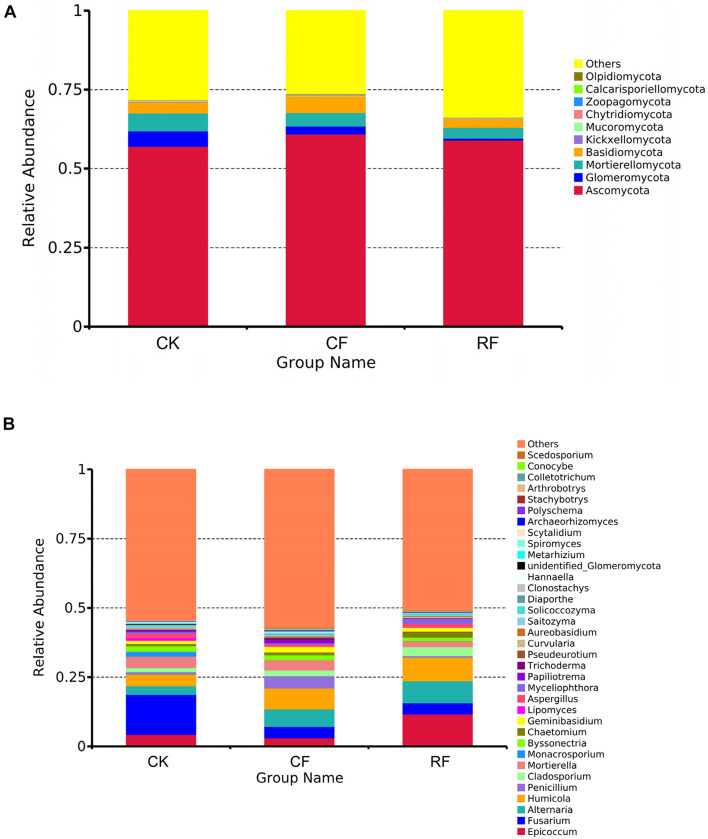
Relative abundance of the fungal phyla **(A)** and genera **(B)** in the rhizospheric soil of cassava with fertilization. CK, CF, and RF indicate without fertilizer applied, with compound fertilizer applied, and with reduced fertilizer applied, respectively.

At the phylum level (Figure 2A), Ascomycota, Basidiomycota, Glomeromycota, and Mortierellomycota were dominant in rhizospheric soil. Among them, Ascomycota was the most abundant, accounting for 57.03%, that increased after fertilizers were applied. The proportion of Basidiomycota was second to Ascomycota and also significantly increased after CF application (*p* ≤ 0.05; Kruskal–Wallis test). However, the proportion of Basidiomycota decreased with RF application. The proportion of Glomeromycota and Mortierellomycota was also decreased after CF and RF application. Compared to CK, the proportion of Glomeromycota in rhizospheric soil with CF and RF application decreased by 49.39 and 87.89%, respectively.

At the genus level (Figure 2B), the dominant genera in cassava rhizospheric soil (top 15) were *Epicoccum*, *Fusarium*, *Alternaria*, *Humicola*, *Penicillium*, *Cladosporium*, *Mortierella*, *Monacrosporium*, *Byssonectria*, *Chaetomium*, *Lipomyces*, *Aspergillus*, *Papiliotrema, Trichoderma*, and *Pseudeurotium*.

The proportion of *Fusarium* showed a decreased tendency after CF or RF application. With CF or RF applied, the proportion of *Chaetomium* gradually increased, indicating that fertilization enhanced the richness of beneficial microorganisms. Notably, the increase in CF or RF applied in rhizospheric soil was about 1.2 and 2.24 times, respectively. Compared to CK, the proportion of *Penicillium* was increased 5.3-fold with CF application.

### Heatmap Analysis of Fungal Community in the Rhizospheric Soil of Cassava

**FIGURE 3:**
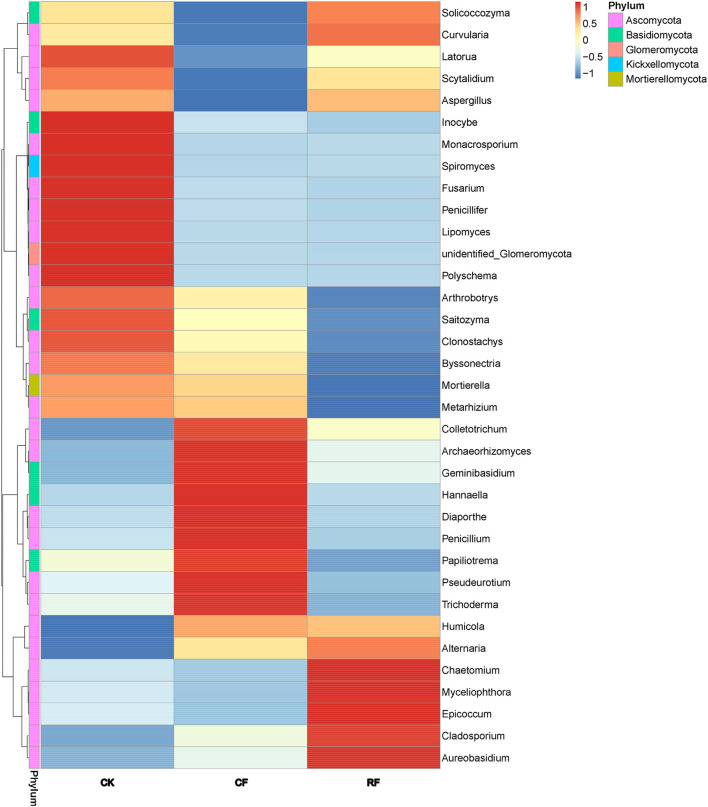
Microbial community heatmap analysis of the fungal genus detected across all of the samples. The relative values for fungal genus are indicated by color intensity with the legend in the upper right of the picture. CK, CF, and RF indicated without fertilizer applied, with compound fertilizer applied, and with reduced fertilizer applied, respectively.

The changes of fungal community structure in the rhizospheric soil of cassava after CF and RF application can be seen more visibly in the relative scale value and color change of the heatmap (Figure 3). At the genus level, the fungal microbial species in CF rhizospheric soil was more, and the species diversity was lower compared to CK. Notably, the fungal microbial species and diversity was lowest among them.

### Venn Analysis of Fungal Community in the Rhizospheric Soil of Cassava

**FIGURE 4:**
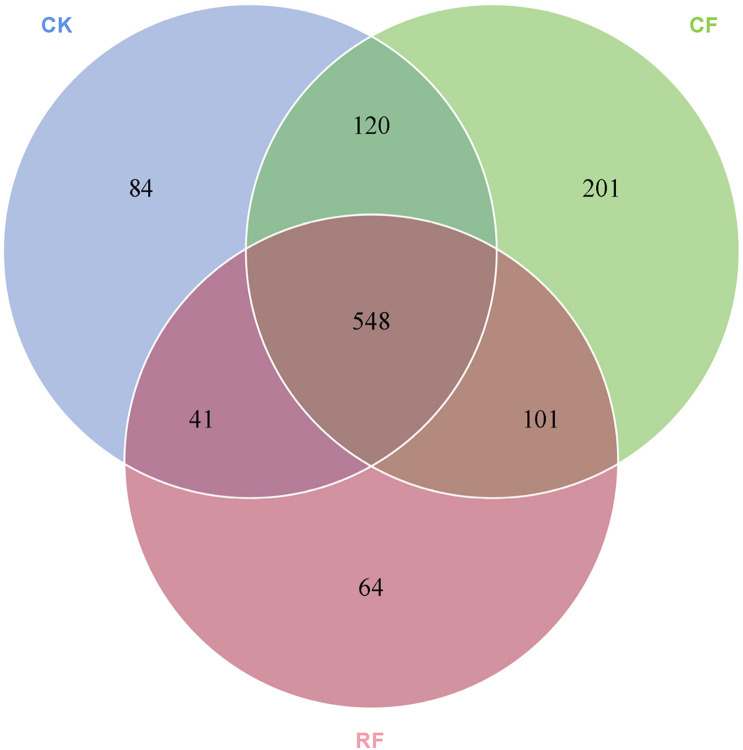
Venn analysis of the number of common and unique operational taxonomic units (OTUs). CK, CF, and RF indicate without fertilizer applied, with compound fertilizer applied, and with reduced fertilizer applied, respectively.

The number of fungal OTUs shared by CK, CF, and RF samples was 548 (Figure 4). The unique OTUs in each sample were relatively small. In the CK, CF, and RF applied samples, the unique OTUs were 84, 201, and 64, respectively. The unique OTUs showed an increasing trend with CF but a decreasing trend with RF, which was consistent with the changing trend of the total OTUs (Table 3). These results showed that fertilization caused changes in the fungal community structure of cassava rhizospheric soil.

### Principal Coordinate Analysis and Cluster Analysis of Fungi in the Rhizospheric Soil of Cassava

**FIGURE 5:**
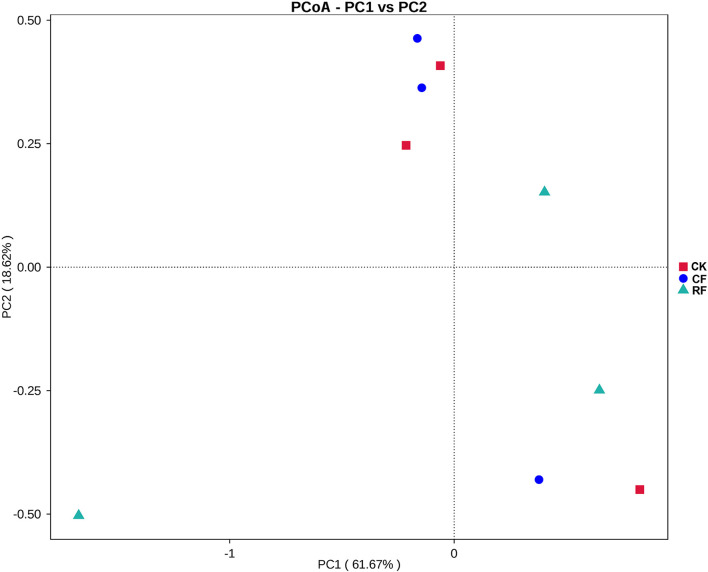
Principal coordinate analysis of operational taxonomic units (OTUs) with different fertilization. CK, CF, and RF indicated without fertilizer applied, with compound fertilizer applied, and with reduced fertilizer applied, respectively.

PCoA analysis of CF and RF rhizospheric soil fungi was performed (Figure 5). The two main coordinates extracted explained 80.29% of the variation, of which PC1 explained 61.67% of the variation and PC2 explained 18.62% of the variation. It showed that the distribution of samples of CK was relatively discrete, and the samples moved further apart. A similar trend was also observed for CF and RF soil samples, indicating that the fungal community structure of rhizospheric soil changed with CF and RF.

**FIGURE 6:**
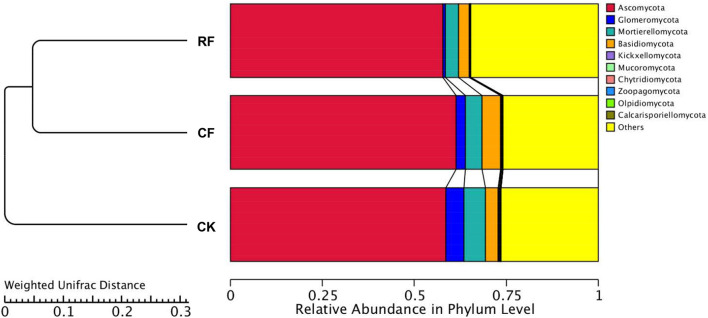
Results of weighted unifrac cluster analysis of operational taxonomic units in the rhizospheric soil of cassava with different fertilization. CK, CF, and RF indicate without fertilizer applied, with compound fertilizer applied, and with reduced fertilizer applied, respectively.

In the cluster analysis (Figure 6), all of the samples clustered into two large groups, among which the samples with fertilization clustered into one group. This finding indicated that fertilization had a certain influence on the fungal community and structure in the rhizospheric soil of cassava.

## Discussion

The high-throughput sequencing approaches using fungal ITS gene marker were beneficial for expanding the current understanding of plant–microbe relationships and comprehensive descriptions of soil fungal community structure and diversity associated with fertilization. To our knowledge, this sequencing technique was used for the first time to analyze the progress of soil fungal community structure and diversity of cassava rhizospheric soil after different fertilizations. It was found that the richness and diversity of fungi in the rhizospheric soil was reduced by RF application. These findings were consistent with the results of a previous study on fertilization effects on the changes in fungal community structure in the rhizospheric soil of *Eucalyptus* and cotton (Chen et al., 2020; Wang et al., 2020).

Ascomycota and Basidiomycota were the dominant phyla in rhizospheric soil fungi of cassava after fertilization, consistent with the results of previous studies (Nie et al., 2018; Chen et al., 2020). Ascomycota has an important role in the degradation of organic matter in rhizospheric soil (Schoch et al., 2009), and a decrease in its amount may impact soil fertility. In the present study, the abundance of Ascomycota increased with CF application, which resulted in a decrease in the amount of SOM, which further affected the amount of soil nutrients in CF (Table 1).

The composition and diversity of fungal community structure play an important role in the balance of the ecosystem (Gao et al., 2019). In addition, many fungi are closely related to plant disease. In this study, we found that the dominant genera (Top 10) in cassava rhizospheric soil were *Epicoccum*, *Fusarium*, *Alternaria*, *Humicola*, *Penicillium*, *Cladosporium*, *Mortierella*, *Monacrosporium*, *Byssonectria*, and *Chaetomium.* At the species level, the proportion of *F. solani* was significantly decreased after fertilization (Supplementary Figure 1; *p* ≤ 0.05, Kruskal–Wallis test). *F*. *solani* is a harmful fungus that causes plant diseases and then affects plant growth and production. The cassava root rot has been responsible for major losses in cassava production, which is mainly caused by the fungi *F. solani* (Barros et al., 2014; Boas et al., 2016). In contrast, the proportions of *Chaetomium* and *Penicillium* were gradually increased after CF application, which are beneficial fungi with biocontrol effect on many plant pathogens (De Cal et al., 2008; Shanthiyaa et al., 2013). Combined with yield data (Table 2), the yields of CF and RF treatments were significantly increased. We inferred that different fertilization patterns altered the fungal community structure of cassava rhizospheric soil; the reduction in the abundance of harmful fungi in the rhizospheric soil can alleviate the incidence rate of cassava root rot. The increase in the abundance of potentially beneficial fungi in the rhizospheric soil can result in promoting plant growth and development, thereby offering a favorable environment for the growth of cassava, consequently increasing yield and quality of cassava. These findings indicated that there is a positive correlation between fungal community structure and plant yield.

In this study, the classification, richness, and diversity of fungi were obtained, and then functional prediction was annotated by FunGuild (Clarke and Ainsworth, 1993). The results of FunGuild indicated that most fungi were classified into pathotrophs, symbiotrophs, and saprotrophs (Supplementary Figure 2). It is worth mentioning that the relative abundance of arbuscular mycorrhizal fungi (AMF) in symbiotrophs was significantly decreased after fertilization (*p* ≤ 0.05; Supplementary Figure 3), consistent with the result of a previous study: long-term fertilization or high fertilizer inputs reduced the richness of Glomeromycota (Liu et al., 2012). Therefore, these findings indicated that fertilization reshaped the fungal community structure in the rhizospheric soil of cassava.

Based on PCoA and cluster analysis, it was found that the rhizospheric soil fungi community structure of cassava was classified into two different groups after fertilization (Figure 6), indicating that fertilization had a certain influence on the rhizospheric soil fungus community structure (Yu et al., 2017).

The correlation between fungal community structure and environmental physicochemical parameters was analyzed based on the canonical correspondence analysis (CCA). These soil characteristics had been previously described (Table 1). The result of CCA indicated that the two main coordinates extracted explained 91.84% of the variation, of which CCA1 explained 81.99% of the variation and CCA2 explained 9.85% of the variation (Supplementary Figure 4). Organic matter (OM) and available P, K, and N were positively correlated with each other. Moreover, available P, K, and OM showed the greatest influence on the microbial community. These findings indicated that there was a positive correlation between soil nutrition and fungal community structure after fertilization.

In general, the changes in microbial community structure in rhizospheric soil are crucially attributed to fertilization of cassava. This study provides a theoretical basis for cassava cultivation and guides agricultural practices. This will also become an important aspect in improving the management of cassava planting. In the future, the development of special microbial fertilizers or soil amendments for cassava cultivation, to promote plant development, growth, and production, is needed.

## Conclusion

In conclusion, fertilization led to the most dominant fungi phylum Ascomycota increased significantly and the fungi diversity and richness were changed in rhizospheric soil of cassava. In addition, different fertilization patterns altered the fungal community structure of cassava rhizospheric soil, such that the number of potentially beneficial fungi increased, while that of potentially pathogenic fungi decreased, thereby offering a favorable environment for the growth of cassava and thus increasing its yield and quality. Furthermore, available P, K, and OM showed the greatest influence on the microbial community. There was a positive correlation between soil nutrition and fungal community structure after fertilization. Therefore, during actual production, we should give more attention to maintaining the stability of cassava’s rhizospheric soil micro-ecology. In the future, it may become a new trend to use specific microbial fertilizer of cassava for its planting and production.

## Data Availability Statement

The datasets presented in this study can be found in online repositories. The names of the repository/repositories and accession number(s) can be found below: https://www.ncbi.nlm.nih.gov/, SRR12667751.

## Author Contributions

JC, JZ, KL, WO, and SC designed the experiment. JC, JZ, YD, SY, HL, ZY, and WO performed the experiment. JC and JZ wrote the manuscript. All authors analyzed the data and reviewed the manuscript.

## Conflict of Interest

The authors declare that the research was conducted in the absence of any commercial or financial relationships that could be construed as a potential conflict of interest.

## Publisher’s Note

All claims expressed in this article are solely those of the authors and do not necessarily represent those of their affiliated organizations, or those of the publisher, the editors and the reviewers. Any product that may be evaluated in this article, or claim that may be made by its manufacturer, is not guaranteed or endorsed by the publisher.

## Footnotes

1
http://drive5.com/uparse/

2
http://www.drive5.com/muscle/
