# Supplementary figures and images for: Different Fertilizers Applied Alter Fungal Community Structure in Rhizospheric Soil of Cassava (Manihot esculenta Crantz) and Increase Crop Yield
Source: Front Microbiol. 2021 Nov 10;12:663781. doi: 10.3389/fmicb.2021.663781 (PMC8631426; doi:10.3389/fmicb.2021.663781)

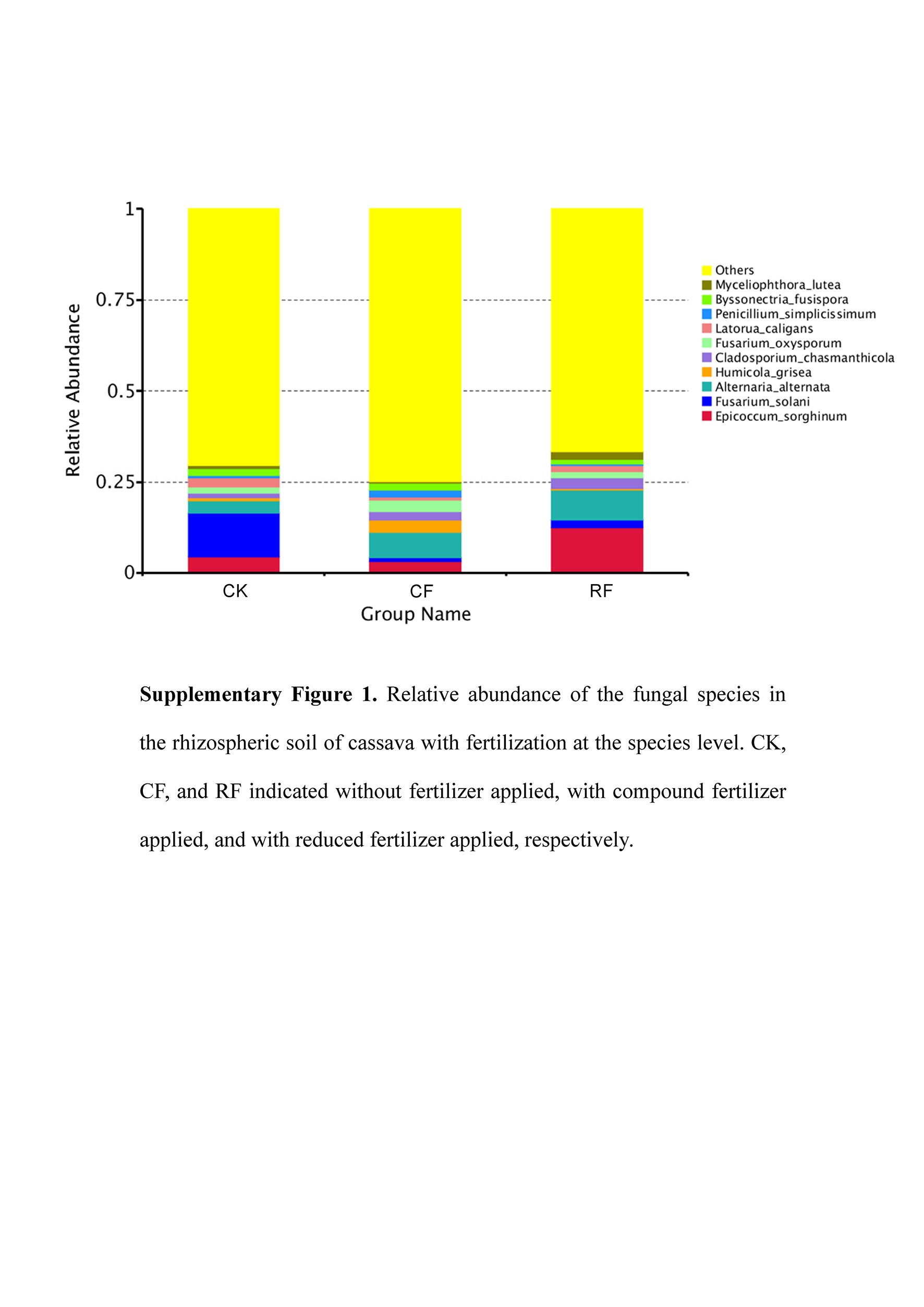

Supplement: Supplementary file 1 [file Image_1.tiff]

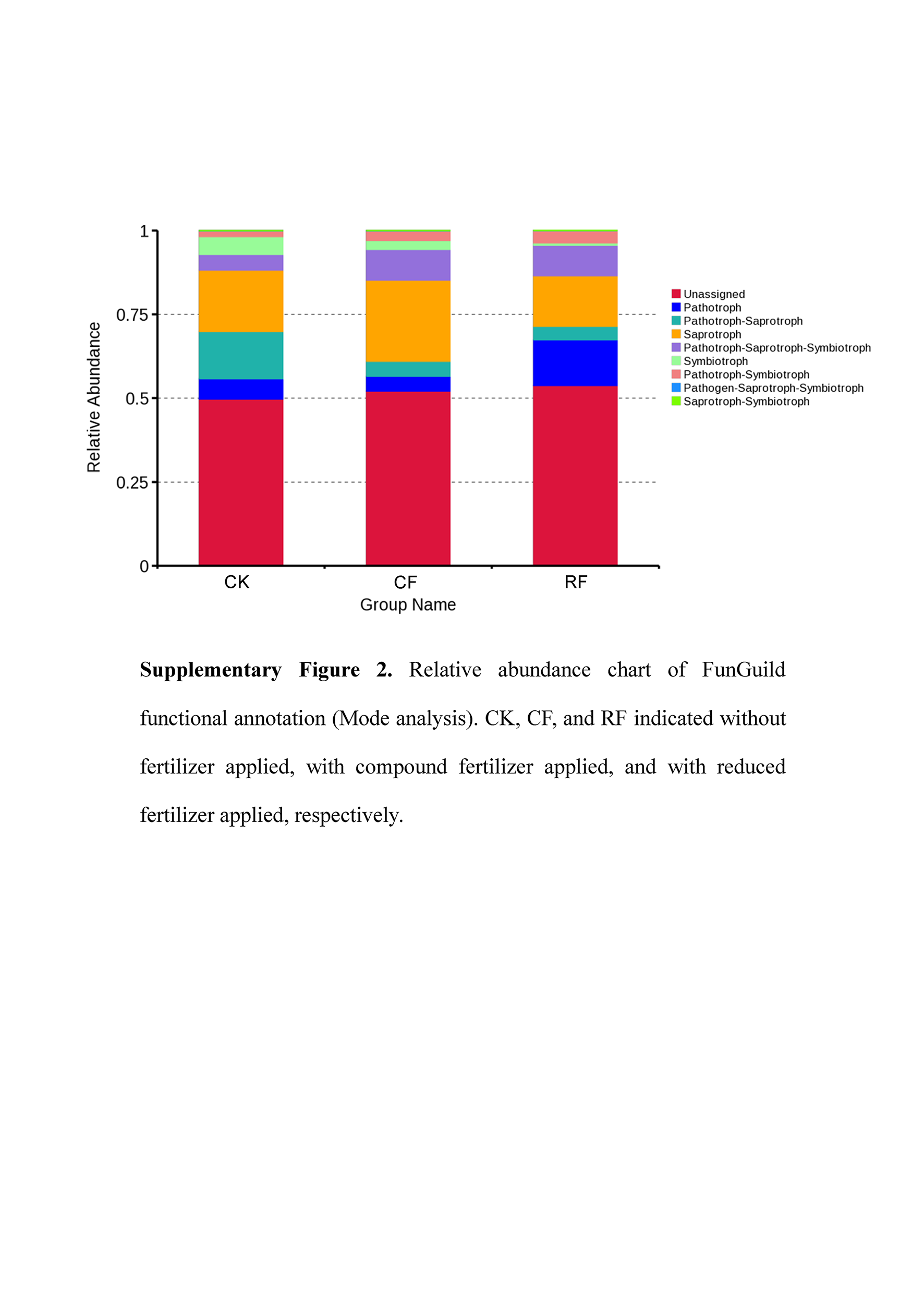

Supplement: Supplementary file 2 [file Image_2.tif]

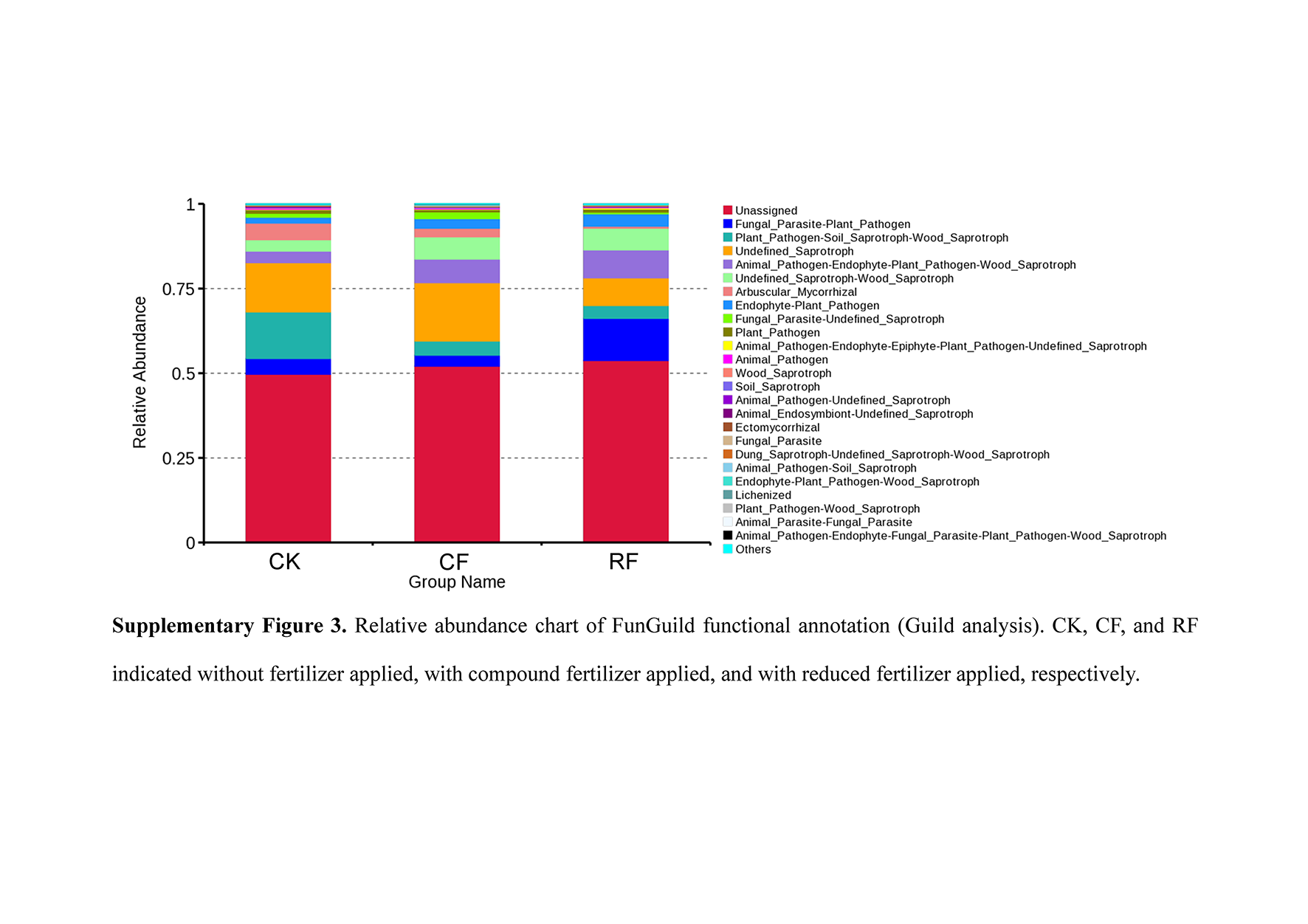

Supplement: Supplementary file 3 [file Image_3.tiff]

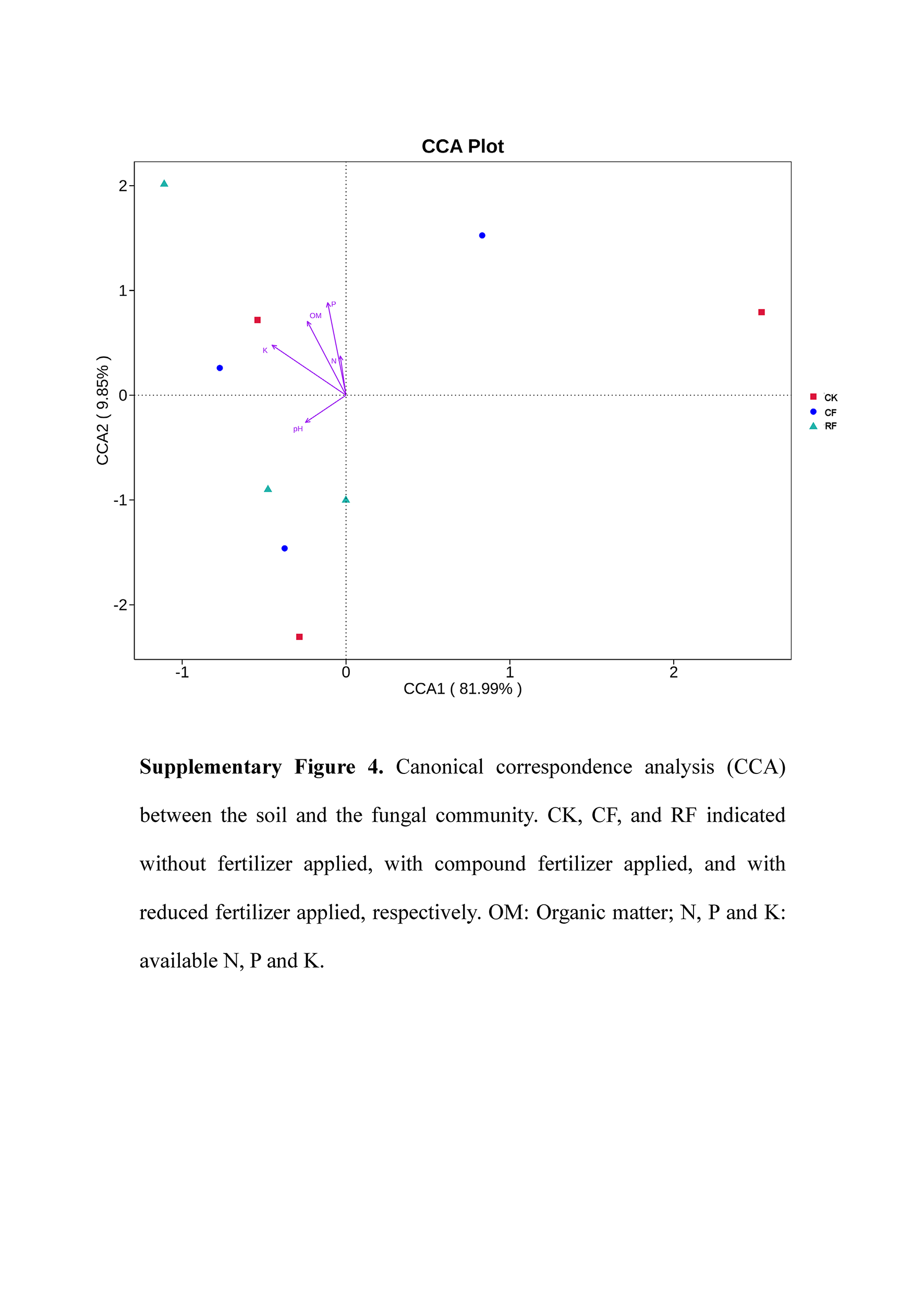

Supplement: Supplementary file 4 [file Image_4.tif]
